# Effects of adding L-arginine orally to standard therapy in patients with COVID-19: A randomized, double-blind, placebo-controlled, parallel-group trial. Results of the first interim analysis

**DOI:** 10.1016/j.eclinm.2021.101125

**Published:** 2021-09-09

**Authors:** Giuseppe Fiorentino, Antonietta Coppola, Raffaele Izzo, Anna Annunziata, Mariano Bernardo, Angela Lombardi, Valentina Trimarco, Gaetano Santulli, Bruno Trimarco

**Affiliations:** a COVID-19 Division, A.O.R.N. Ospedali dei Colli, Naples, Italy; b Department of Advanced Biomedical Sciences, *“Federico II”* University, Naples, Italy; c Department of Medicine, Fleischer Institute for Diabetes and Metabolsim (*FIDAM*), Einstein - Mount Sinai Diabetes Research Center *(ES-DRC)*, Albert Einstein College of Medicine, New York, NY, USA; d Department of Microbiology and Immunology, Albert Einstein College of Medicine, New York, NY, USA; e Department of Neuroscience, Reproductive Sciences, and Dentistry, *"Federico II"* University, Naples, Italy; f Department of Molecular Pharmacology, Wilf Family Cardiovascular Research Institute, Einstein Institute for Aging Research, Albert Einstein College of Medicine, New York, NY, USA; g International Translational Research and Medical Education (*ITME*) Consortium, Naples, Italy

**Keywords:** COVID-19, Endothelial dysfunction, SARS-CoV-2, Arginine, Clinical trial, Coronavirus, Immune response

## Abstract

**Background:**

We and others have previously demonstrated that the endothelium is a primary target of the severe acute respiratory syndrome coronavirus 2 (SARS-CoV-2), and L-arginine has been shown to improve endothelial dysfunction. However, the effects of L-arginine have never been evaluated in coronavirus disease 2019 (COVID-19).

**Methods:**

This is a parallel-group, double-blind, randomized, placebo-controlled trial conducted on patients hospitalized for severe COVID-19. Patients received 1.66 g L-arginine twice a day or placebo, administered orally. The primary efficacy endpoint was a reduction in respiratory support assessed 10 and 20 days after randomization. Secondary outcomes were the length of in-hospital stay, the time to normalization of lymphocyte number, and the time to obtain a negative real-time reverse transcription polymerase chain reaction (RT-PCR) for SARS-CoV-2 on nasopharyngeal swab. This clinical trial had been registered at ClinicalTrials.gov, identifier: NCT04637906.

**Findings:**

We present here the results of the initial interim analysis on the first 101 patients. No treatment-emergent serious adverse events were attributable to L-arginine. At 10-day evaluation, 71.1% of patients in the L-arginine arm and 44.4% in the placebo arm (*p* < 0.01) had the respiratory support reduced; however, a significant difference was not detected 20 days after randomization. Strikingly, patients treated with L-arginine exhibited a significantly reduced in-hospital stay *vs* placebo, with a median (interquartile range 25^th^,75^th^ percentile) of 46 days (45,46) in the placebo group *vs* 25 days (21,26) in the L-arginine group (*p* < 0.0001); these findings were also confirmed after adjusting for potential confounders including age, duration of symptoms, comorbidities, D-dimer, as well as antiviral and anticoagulant treatments. The other secondary outcomes were not significantly different between groups.

**Interpretation:**

In this interim analysis, adding oral L-arginine to standard therapy in patients with severe COVID-19 significantly decreases the length of hospitalization and reduces the respiratory support at 10 but not at 20 days after starting the treatment.

**Funding:**

Both placebo and L-arginine were kindly provided by Farmaceutici Damor S.p.A., Naples

**Research in context**

**Evidence before this study**

The severe acute respiratory syndrome coronavirus 2 (SARS-CoV- 2) infection has spread worldwide, causing disease and mortality, as well as social disruption and economic loss. Endothelial cells have been shown to be a cardinal target in COVID-19, and L-arginine is known to improve endothelial dysfunction. PubMed and medRxiv preprint searches were updated on June 24, 2021 using the search criteria "SARS-CoV-2" "COVID-19" “L-arginine”. These searches retrieved no items.

**Added value of this study**

The first interim analysis of this randomized, double-blind, placebo-controlled, parallel-group trial shows that patients hospitalized for severe COVID-19 treated with L-arginine displayed a significantly reduced in-hospital stay *vs* placebo (log-rank *p* <  0.0001). Furthermore, at the 10-day evaluation, 71.1% of patients in the L-arginine group and 44.4% in the placebo group (*p* < 0.01) had reduced the respiratory support; however, a significant difference was not detected 20 days after randomization, most likely because most of the participants in the L-arginine arm had been already discharged from the hospital by this time.

**Implications of all the available evidence**

There is concrete evidence that endothelial dysfunction is a fundamental feature of COVID-19. However, no clinical trial has actually shown that intervention improving endothelial function could be beneficial in COVID-19. Our interim findings indicate for the first time that adding L-arginine orally to standard therapy in patients with severe COVID-19 significantly reduces the length of hospital stay and respiratory support.

Alt-text: Unlabelled box

## 1 Introduction

The clinical course of coronavirus disease 2019 (COVID-19) consists of two main phases: viral infection and immune/inflammatory response, requiring distinct therapeutic approaches to stop virus replication and to attenuate the inflammatory state that is commonly observed in COVID-19 patients and may contribute to multiorgan failure [1], [2], [3], [4]. Although several approved drugs and investigational agents have shown some activity against severe acute respiratory syndrome coronavirus 2 (SARS-CoV-2), the treatment of COVID-19 remains a critical challenge [5], [6], [7], [8], [9], [10], [11], [12], [13].

Clinical and preclinical evidence supports the view that the endothelium is a key target organ in COVID-19, providing a mechanistic rationale behind its systemic manifestations [14]. Hence, COVID-19 can be considered a systemic vascular disease affecting multiple organs due to endothelial damage [14,15]. More recently, amino acid metabolism has been shown to be a crucial factor in the pathophysiology of COVID-19 [16]; specifically, decreased plasma L-arginine levels along with enhanced arginase activity have been reported in COVID-19 patients, especially in the most severe forms [17,18].

On these grounds, given the well-established beneficial effects of L-arginine on endothelial function [19], we hypothesized that oral L-arginine supplementation could be helpful for contrasting the inflammatory state in COVID-19. Therefore, we designed the present single-center double-blind randomized, placebo-controlled trial with a parallel group scheme, to test the hypothesis that, compared to placebo, the addition of oral L-arginine to the hospital standard therapy is an efficacious treatment for patients hospitalized for COVID-19.

## 2 Methods

### 2.1 Study design and participants

We designed an investigator-initiated, individually randomized, placebo-controlled, double-blind trial aiming to assess the effectiveness and safety of oral L-arginine in adults (aged ≥ 18 years) admitted to hospital with severe COVID-19, defined as previously reported by other investigators [15,20]. The trial (registered at ClinicalTrials.gov, Identifier: NCT04637906) was conducted at the *Domenico Cotugno* Hospital (Naples, Italy). The study protocol is reported in Appendix 1.

Ethical approval was obtained from the institutional Ethical Committee of the Hospital. Written informed consent was collected from all patients, or their legal representative if they were unable to provide consent. The trial was performed in accordance with the principles of the Declaration of Helsinki and the International Conference on Harmonization-Good Clinical Practice guidelines.

Eligible hospitalized patients were men and non-pregnant women with COVID-19 who were at least 18-year-old and were RT-PCR positive for SARS-CoV-2; patients were screened by applying the following eligibility criteria.

### 2.2 Inclusion criteria

Presence of all the following conditions:

• Pneumonia confirmed by chest imaging;
• Oxygen saturation of 93% or lower on room air;
• Ratio of alveolar oxygen partial pressure to fractional inspired oxygen (PaO_2_/FiO_2_, or P/F) of 300 or less;
• Lymphocytopenia, defined as lymphocytes < 1500/μL or < 20% of white blood cells.

**Exclusion criteria:**

• History of L-arginine intolerance;
• Diagnosis of chronic pulmonary disease - currently under treatment;
• Pregnancy or breastfeeding;
• Neutropenia due to neoplasms of the hematopoietic system or other organs with invasion of the bone marrow;
• Use of immunosuppressive drugs, other than corticosteroids, or cytotoxic chemotherapy within the previous three weeks;
• Symptoms onset > 15 days before enrollment;
• Enrollment into an investigational treatment study for COVID-19 in the 30 days before screening;
• Refusal to provide written informed consent.

### 2.3 Procedures

Eligible patients were assigned, with a 1:1 ratio, based on a computer-generated randomization table, to add to the hospital standard therapy 1 bottle containing 1.66 g of L-arginine (Bioarginina®, Farmaceutici Damor S.p.A.) or 1 bottle of identical aspect not containing L-arginine, twice a day orally for the whole hospitalization period. The formulation of Bioarginina® used in this study consists of oral vials containing 1.66 g of L-arginine, whose taste is fully buffered by the presence of sucrose and by an acidity regulator (anhydrous citric acid); therefore, there were no differences in appearance, smell, or taste between treatment and placebo. The dose was chosen based on a previous study proving that such a regimen was safe and significantly effective in ameliorating the oxidative metabolism of professional water polo players [21]. Respiratory support was defined as follows (from the more to the less intense one): NIV: non-invasive ventilation; CPAP: continuous positive airway pressure; HFNC: high-flow nasal cannula; LTOT: Long-term oxygen therapy [22]. Since symptom duration has been shown to have an effect on prognosis [13], the actual duration of symptoms (≤ 8-days *vs* > 8-days) was taken into account during randomization. Protocol adherence was assessed daily during the hospitalization period. Patients, ward doctors, trial personnel and outcome assessors were unaware of the type of supplementation provided. All clinical interventions, including the use of antibiotics, corticosteroids, anticoagulants, ventilation strategy, and laboratory investigations were at the discretion of the treating physicians according to clinical needs for both treatment groups.

### 2.4 Clinical and laboratory parameters

A single follow-up form was completed by the trial staff when each patient was discharged or had died, whichever occurred first. Information was recorded regarding the patients’ adherence to the assigned treatment, administration of other treatments, duration of symptoms on admission, duration and type of respiratory support, renal dialysis or hemofiltration. In addition, we obtained routine health care and registry data, including information on vital status (or date and cause of death), discharge from the hospital, as well as respiratory and renal support therapy. The P/F ratio was calculated as previously described [23,24], obtaining PaO_2_ from arterial blood gas test; FiO_2_ was considered 0.21 when the patient was breathing room air [25].

Before randomization, the demographic characteristics and other clinical and laboratory data required by the routine diagnostic activity were collected. These parameters were evaluated daily during the hospitalization period according to routine hospital practice. The safety assessment included daily monitoring for adverse events, clinical laboratory testing, 12-lead electrocardiogram, and daily measurement of vital signs.

### 2.5 Objectives of the study

The primary clinical endpoint was a reduction in respiratory support (meaning a transition to a less intense assistance, NIV → CPAP → HFNC → LTOT → Room air), evaluated 10 and 20 days after randomization.

Secondary outcomes were the length of in-hospital stay, the time to lymphocyte number normalization, and the time to obtain a negative RT-PCR for SARS-CoV-2 on nasopharyngeal swab.

Safety outcomes included treatment-emergent adverse events, serious adverse events, and premature discontinuations of study drug.

### 2.6 Statistical analysis

Since no reliable data were available for an accurate calculation of the sample size, we harnessed data from our preliminary clinical experiences conducted with the administration of L-arginine in COVID-19 patients and those of a historical group of COVID-19 patients undergoing the same therapeutic regimen except the daily oral supplementation of L-arginine. Based on these data, we hypothesized a difference of at least 35% in the primary outcome between the two study groups. According to this hypothesis, we determined that it was necessary to enroll a population of 290 patients to detect a statistically significant difference with a 2-side α level of 0.05 and a power of 80%.

Two interim analyses were planned for the assessment of efficacy and tolerability after completion of the first 100 and 200 patients. The study discontinuation threshold was set at *p* < 0.01 for efficacy and *p* < 0.01 for tolerability. Enrollment was planned to continue until the Data and Safety Monitoring Board (DSMB) recommended stopping the trial for evidence of efficacy, futility, or harm, based on evaluation of all the available data, including data internal and external to the trial.

The characteristics of the participants are reported as the mean ± standard deviation (SD) for approximately normally distributed continuous variables, as the median (interquartile range [IQR]: 25^th^ percentile, 75^th^ percentile) for severely skewed continuous variables and as numerical values (percentages) for categorical variables. Normal distribution of the data was verified via normal probability plots and confirmed with the skewness/kurtosis test for normality. Bivariate tests were used to assess the association between the treatment with L-arginine and the baseline characteristics and treatments of the patients included in the study. Statistical significance was determined by a *p* value < 0.05. In the statistical analysis, differences for continuous variables were evaluated using two-sample *t*-test for approximately normally distributed variables and Mann-Whitney *U* test for severely skewed variables. Chi-square or Fisher tests were used to measure associations between dichotomous and categorical variables.

Multivariable logistic regression analysis was performed in order to investigate the association between L-arginine treatment and the primary outcome (reduction in respiratory support), adjusting for potential confounders; a backward selection method was applied to create the final model. Log-rank test was used to compare the length of in-hospital stay between the L-arginine and the placebo groups and a Cox proportional hazards regression analysis was employed to relate L-arginine treatment to length of hospitalization adjusting for likely confounders. All analyses were performed using SPSS 26.0.

### 2.7 Role of the funding source

Both placebo and L-arginine were kindly provided by Farmaceutici Damor S.p.A., Naples, Italy, which had no role in the design and conduct of the study, collection, management, analysis, and interpretation of the data, preparation, review, or approval of the manuscript, and decision to submit the manuscript for publication. GF, RI, and BT had full access to all data in the study and take final responsibility for manuscript submission.

## 3 Results

**Fig. 1:**
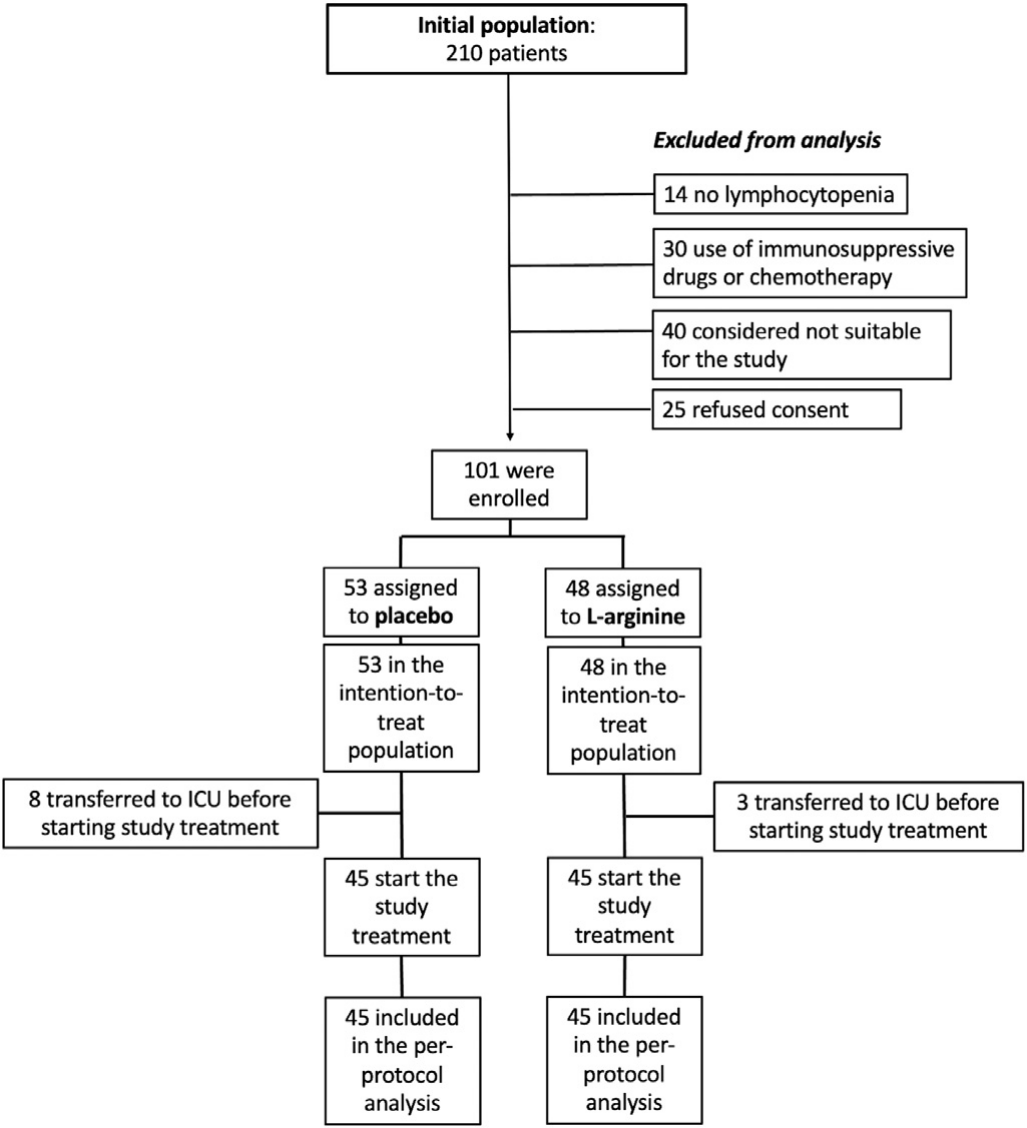
Study flowchart.

Between November 2020 and April 2021, 210 patients were screened, of whom 101 were eligible (Fig. 1); 48 patients were assigned to receive L-arginine and 53 to receive placebo; no patient in either group withdrew her/his previously given written informed consent after randomization, so 48 and 53 patients were included in the intention-to-treat (ITT) population for the active and the placebo treatment, respectively. Patients were randomized and received study treatments with a mean of 7.8 days after symptoms onset. At randomization, all patients were receiving oxygen, with or without non-invasive ventilation. Since 8 patients randomized to receive placebo and 3 in the L-arginine arm were transferred to the intensive care unit (ICU) before starting the study, we had in the per protocol (PP) analysis a total of 90 patients who started and successfully completed the efficacy and safety evaluation period (Fig. 1). A 100% protocol adherence was achieved for this interim analysis. The demographic, anthropometric, and clinical characteristics of the 90 patients are shown in Table 1. The most common comorbidity in both arms was hypertension, followed by coronary heart disease, and diabetes. There was no difference in the number of days between onset of symptoms and starting treatment in patients of the control group and those in the L-arginine group.

**Table 1:** Baseline characteristics and treatments of the per-protocol analysis population. Data are presented as mean ± SD for continuous variables normally distributed and as median (IQR: 25^th^ percentile, 75^th^ percentile) for non-normally distributed continuous variables; percentages are reported for categorical variables. Parameters in bold denote a significant difference (*p* < 0.05). ALT: Alanine aminotransferase; AST: aspartate aminotransferase; BUN: blood urea nitrogen; CPAP: continuous positive airway pressure; CRP: C Reactive Protein; HFNC: high-flow nasal cannula; LMWH: low molecular weight heparin; LTOT: long-term oxygen therapy; NIV: non-invasive ventilation.

	*Placebo (n = 45)*	*L-arginine (n = 45)*
Gender (M/W) (%)	86.7/13.3	75.6/24.4
**Age (years)**	65.9 ± 11.7	57.4 ± 13.2
Hypertension (%)	42.2	31.1
Coronary artery disease (%)	17.8	11.1
Smokers (%)	8.9	6.7
Obesity (%)	11.1	8.9
Diabetes (%)	8.9	11.1
Time between onset of symptoms and admission (days)	7 (6, 10)	6.5 (5, 10)
White blood cells (n/μL)	10319.7 ± 5532.7	9210.0 ± 3310.0
Lymphocytes (n/μL)	8.5 (4.8, 12.6)	7.3 (5.1, 10.4)
CRP (mg/L)^a^	8.2 (4.8, 13.05)	5.3 (3.1, 13)
D-dimer (ng/mL)	397 (219, 1540)	256 (183, 441)
AST (U/L)	31 (26, 50)	34 (26, 42)
ALT (U/L)	42 (31, 62)	41 (33.5, 60.5)
Creatinine (mg/dL)	0.9 (0.75, 1.1)	0.8 (0.7, 0.9)
BUN (mg/dL)^a^	73.0 ± 45.6	58.1 ± 25.0
Sodium (mmol/L)^b^	137.5 ± 4.5	137.8 ± 4.4
Potassium (mmol/L)^b^	4.6 ± 0.9	4.4 ± 0.5
Asthenia (%)	91.1	93.3
Dyspnea (%)	95.6	95.6
Cough (%)	33.3	28.9
Fever (%)	75.6	73.3
Sputum (%)	8.9	2.2
PaO_2_ (kPa)	10.4 ± 1.39	9.92 ± 1.4
PaCO_2_ (kPa)	4.6 ± 0.6	5.09 ± 0.6
P/F (PaO_2_/FiO_2_)	154.6 ± 49.5	161.7 ± 62.3
Remdesivir (%)	24.4	31.1
LMWH (%)	93.3	91.1
Steroids (%)	100	100
Respiratory support:		
**LTOT (%)**	42.2	20.0
HFNC (%)	73.3	62.2
**CPAP (%)**	2.2	13.3
**NIV (%)**	0	11.1

a available in 43 patients in the placebo group and in 44 patients in the L-arginine group;

b available in 44 patients in both the placebo group and the L-arginine group.

**Fig. 2:**
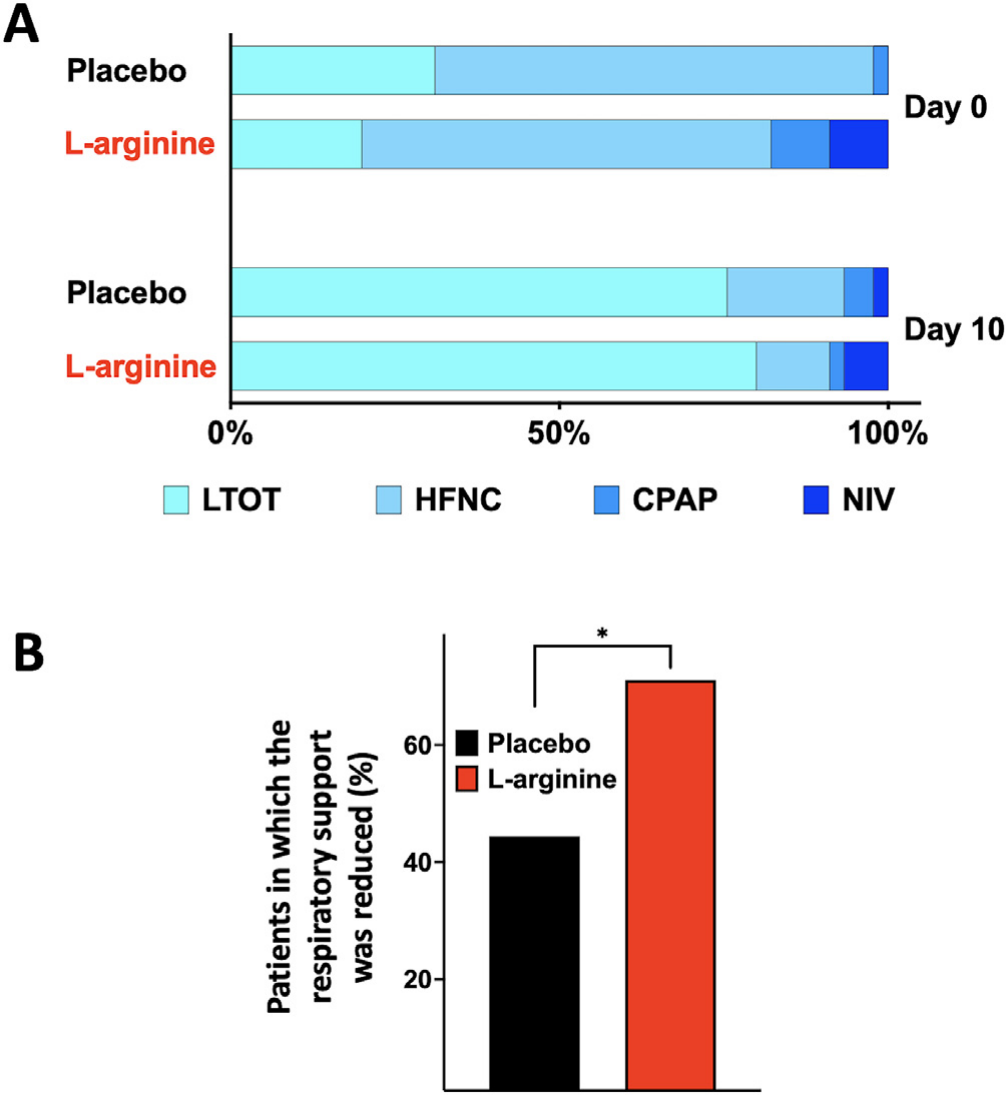
Respiratory support at baseline and at day 10 (**A**) and percentage of patients in which the respiratory support was reduced, evaluated 10 days after randomization (**B**) in the per-protocol analysis; *: *p* < 0.01. CPAP: continuous positive airway pressure; HFNC: high-flow nasal cannula; LTOT: long-term oxygen therapy; NIV: non-invasive ventilation.

The primary clinical endpoint was the reduction in respiratory support (according to the sequence NIV → CPAP → HFNC → LTOT → Room Air) assessed 10 and 20 days after randomization. At 10-day evaluation, 44.4% in the placebo group and 71.1% in the L-arginine group (*p* < 0.01) had reduced the respiratory support (Fig. 2).

A multivariable logistic regression was performed in order to examine the magnitude of association between the treatment with L-arginine and the primary outcome and to better identify explanatory variables that were associated with a reduction of the respiratory support (Table 2). When adjusting the main analysis for likely confounders including age, gender, symptom duration, hypertension, diabetes, treatment with remdesivir or heparin, and values of creatinine, lymphocytes, and D-dimer, the odds of having a reduction in respiratory support were 6.6-fold higher in those who were in the treatment group compared with those in the placebo group (OR, 6.62; 95% CI, 1.55, 28.22; *p* = 0.01).

**Table 2:** Multivariable logistic regression analysis assessing in the per-protocol analysis the association of L-arginine with the primary outcome at 10 days. CI: confidence interval; LMWH: low molecular weight heparin; OR: odds ratio; symptom duration before hospital admission was categorized as ≤ 8 and > 8 days.

	OR	95% CI		p
		Lower	Upper	
Age	1.058	0.991	1.129	0.090
Gender	3.449	0.793	15.011	0.099
Symptom duration	0.710	0.199	2.531	0.598
Hypertension	0.590	0.147	2.361	0.456
**Diabetes**	0.011	0.001	0.389	0.013
Creatinine	0.749	0.209	2.683	0.658
Lymphocytes	1.109	0.994	1.238	0.065
D-dimer	1.001	1.000	1.001	0.060
LMWH	0.824	0.090	7.534	0.864
Remdesivir	1.142	0.302	4.323	0.845
**L-arginine**	6.622	1.554	28.223	0.01

On the contrary, 20 days after randomization we failed to detect any significant difference in the primary outcome between the study arms (placebo group: 13 out of 25, 52%; L-arginine group: 4 out of 13, 30.8%; non-significant). Similarly, the P/F ratio was significantly different at 10 days (Placebo: 186.4 ± 68.3 *vs* L-arginine: 228.3 ± 93; *p* = 0.02) but not at 20 days (Placebo: 231.0 ± 93 *vs* L-arginine: 263.5 ± 103).

**Fig. 3:**
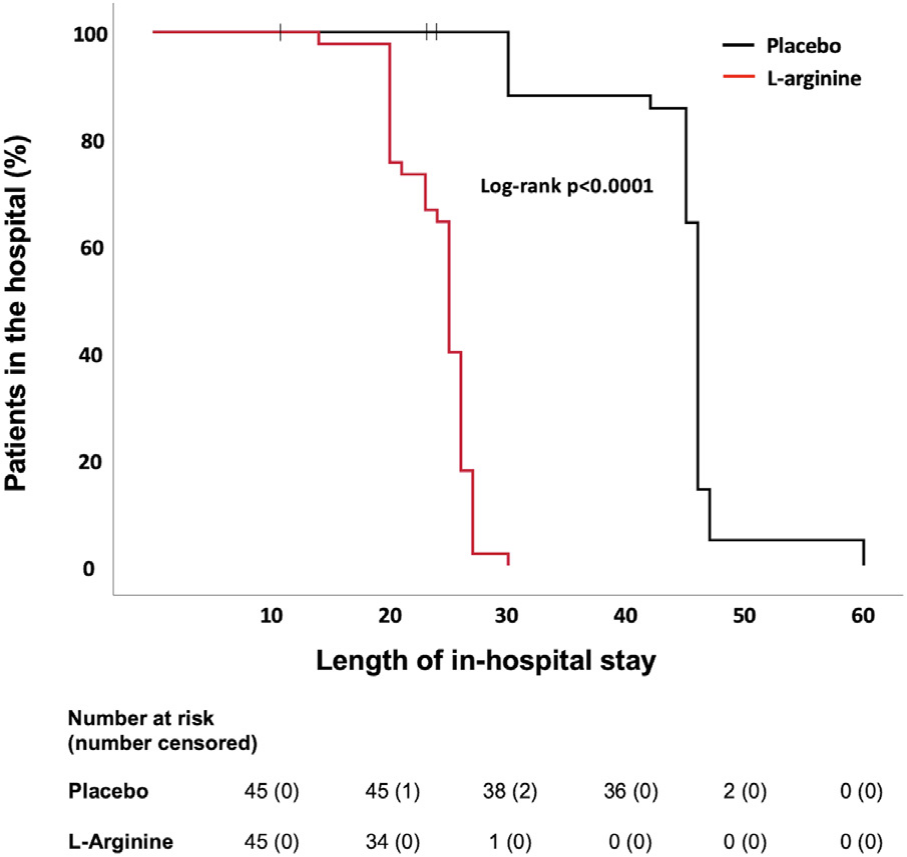
Kaplan-Meier curves assessing in the per-protocol analysis the length of in-hospital stay.

The multivariable logistic regression performed on the ITT population at 10 and 20 days after randomization is presented as supplementary material (Table S1 and Table S2, respectively). Among the secondary outcome measures, differences between active treatment group *vs* placebo group were statistically significant only for the time to hospital discharge, which was significantly shorter in the L-arginine arm (Fig. 3). Specifically, the median time to hospital discharge in the L-arginine group and placebo group was 25 and 46 days, respectively, and the distributions in the two groups differed significantly (*p* < 0.001). This association remained significant in a Cox regression analysis (Table 3) using the fully adjusted multivariable model including age, gender, symptom duration, hypertension, diabetes, treatment with remdesivir or heparin, and values of creatinine, lymphocytes, and *D*-dimer (HR, 41.6; 95% CI, 12.18, 142.10; *p* < 0.0001). The variables used in the regression analyses were selected according to the available literature on COVID-19^7-9,11^.

**Table 3:** Cox proportional hazards model assessing in the per-protocol analysis the effect of L-arginine on length of in-hospital stay, adjusting for potential confounders. CI: confidence interval; HR: hazard ratio; LMWH: low molecular weight heparin; symptom duration before hospital admission was categorized as ≤ 8 and > 8 days.

	HR	95% CI		p
		Lower	Upper	
**Age**	0.971	0.944	0.998	0.035
Gender	0.918	0.463	1.819	0.805
Symptom duration	1.794	0.992	3.245	0.053
Hypertension	0.798	0.441	1.445	0.457
Diabetes	1.244	0.445	3.478	0.678
Creatinine	0.846	0.544	1.315	0.457
Lymphocytes	1.02	0.979	1.064	0.340
D-dimer	0.999	0.999	1.001	0.768
LMWH	0.874	0.355	2.148	0.769
Remdesivir	0.986	0.558	1.744	0.962
**L-arginine**	41.599	12.178	142.102	<0.001

The rate of lymphocyte number normalization was similar between the 2 study arms (17.5% and 24.4% in the placebo group and 36.1% and 20% in the L-arginine group at days 10 and 20, respectively; both not statistically significant). The time to obtain a negative RT-PCR for SARS-CoV-2 on nasopharyngeal swab was comparable between groups (20.4 ± 13.8 days in the placebo group and 24.8 ± 14.5 days in the L-arginine group, *p* = 0.21).

The 11 patients transferred to the ICU (8 randomized to the placebo group, 3 to the L-arginine group) deceased shortly after the transfer, before they could actually start the treatment with L-arginine or placebo. Since these patients were transferred to the ICU right after the randomization, they have been included in the ITT but not in the PP analysis. Besides, 3 other patients in the placebo arm (0 in the L-arginine arm) died after day 20. Thus, the total mortality was 3.3% in the PP analysis (Placebo: 6.7%, L-arginine: 0) and 13.9% in the ITT analysis (Placebo: 20.8%, L-arginine: 6.3%; p = 0.035).

### 3.1 Adverse events

Four serious adverse events were reported (pneumomediastinum, pancreatitis, and pulmonary embolism in the placebo group, pneumothorax in the L-arginine group) which were considered unrelated to the study treatment by the investigators.

## 4 Discussion

In this randomized clinical trial involving adults with severe COVID-19, L-arginine plus standard care compared with standard care alone significantly reduced the need for respiratory support and, foremost, the duration of hospitalization. L-arginine was not associated with increased risk of adverse events in these critically ill COVID-19 patients. Notably, the safety of oral L-arginine had been previously ascertained in other trials [26,27].

We selected the reduction of respiratory support as the primary endpoint because it comprises both pathophysiologic and clinical determinants. Thus, in order to assess the role of L-arginine supplementation in the reduction of respiratory support, we performed a multivariate analysis which demonstrated that treatment with L-arginine was the main determinant of this phenomenon, ruling out the possibility that it could be merely due to the younger age of this group of patients. This conclusion is corroborated by the observation that L-arginine supplementation was the main determinant in the reduction of in-hospital stay duration; the in-hospital stay median (IQR 25^th^,75^th^ percentile) number of days was 46 (45,46) in the placebo group *vs* 25 (21,26) in the L-arginine group (*p* < 0.0001, Mann-Whitney); this finding was also confirmed after adjustment for potential confounders including age.

On the contrary, there were no significant improvements in the time to normalization of lymphopenia nor in the time to obtain a negative RT-PCR for SARS-CoV-2 on nasopharyngeal swab, two findings that may appear in contrast with a beneficial effect of L-arginine in the treatment of COVID-19. However, L-arginine may exert its favorable effect through means of an improvement of endothelial function without any interference with virus replication, which strongly affects the time to obtain a negative RT-PCR for SARS-CoV-2 on nasopharyngeal swab. Indeed, the currently accepted view of COVID-19 pathogenesis includes a viral tissue injury followed by an inflammatory host immune response, which drives hypercytokinemia and aggressive inflammation, resulting in endotheliitis, increased apoptotic activity, thrombotic events, and intravascular coagulation [14,15,28,29]. The excessive inflammatory response against SARS-CoV-2 is thought to orchestrate disease severity in patients with COVID-19 and is associated with profound lymphopenia and substantial mononuclear cell infiltration in lungs, heart, spleen, lymph nodes, intestine, and kidney, as confirmed in postmortem analyses [4,30]. *In vitro* studies of cell lines as well as immunohistochemichal and electron microscopy analyses of human tissues suggested the presence of SARS-CoV-2 within endothelial cells [31], [32], [33], [34]. Endothelial cell infection with consecutive inflammatory cell recruitment and endothelial dysfunction may explain the impaired microcirculation observed across vascular beds in COVID-19, triggering vasoconstriction, ischemia, and a pro-coagulant state [14]. Therefore, endotheliitis has been suggested as the major cause of systemic impaired microcirculatory function observed in different vascular beds in COVID-19 patients [14]. Our findings are consistent with previous reports indicating that after the early stage the disease may be dominated by immunopathological elements, with active viral replication playing a secondary role [4,15,[35], [36], [37], [38], [39]].

Further supporting our data, L-arginine depletion has been shown to increase the production of reactive oxygen species, exacerbating inflammation [40].

The exact mechanisms linking SARS-CoV-2 infection and lymphopenia remain not fully defined [41], [42], [43]. SARS-CoV-2 is internalized into cells via ACE2, which is widely expressed by cardiopulmonary tissues and certain hematopoietic cells like monocytes and macrophages [44]. Owing to low expression of ACE2 on *T* cells [45], direct viral attack of *T* cells via ACE2 can hardly explain the occurrence of lymphopenia. Alternatively, the reduction in lymphocyte count may be attributed to increased cell apoptosis [46]. Several investigators reported a negative correlation between *T* cell numbers and the concentration of cytokines including interleukin-6 (IL-6), IL-10, interferon-γ (IFN-γ), and tumor necrosis factor-α (TNF-α) in COVID-19 patients [46,47]. Highly dysregulated cytokine release might promote *T* lymphocyte apoptosis by activating extrinsic and intrinsic apoptosis pathways during SARS-CoV-2 infection [48,49]. Whatever the case, the observation that in our study L-arginine is able to shorten the disease duration without reducing the time to normalization of lymphocyte number is not surprising even because the whole study population during hospitalization received glucocorticoids, which in severe COVID-19 patients may promote lymphocyte depletion [9,36,42,50]. The activity of arginase–the enzyme responsible for metabolizing L-arginine to ornithine and urea–could also have a negative impact on *T* cell function, which has been shown to depend on L-arginine supply [51], [52], [53], [54]. *In vitro* assays revealed that *T* cell proliferative capacity is significantly reduced among COVID-19 patients and can be restored through L-arginine supplementation [18]. Tadié and collaborators have demonstrated that COVID-19 patients with severe acute respiratory distress syndrome present an elevated number of myeloid-derived suppressor cells, which was directly correlated to enhanced arginase activity, effectively depleting L-arginine from the microenvironment [18,55]. Ergo, the effects of L-arginine on *T* cell function may be of greater importance than the mere impact on lymphocyte number.

Intriguingly, diabetes was significantly associated with the primary outcome but not with the hospitalization length; this observation could be related to the previously reported detrimental role of diabetes on lung function [56,57]; the pathophysiology of pulmonary disease in diabetic patients is complex and multifactorial and some of the proposed underlying mechanisms include endothelial dysfunction, microangiopathy of alveolar capillaries, oxidative stress, autonomic neuropathy, and alterations of connective tissue [58], [59], [60].

At day 20 after randomization, we did not detect any significant difference in the rate of patients that transitioned to a lower respiratory support; however a nearly twice the number of participants in the L-arginine arm had been already discharged from the hospital by this time, leaving on a small number of patients remaining-likely the sickest patients of the cohort; therefore, we can speculate that many of the patients with earlier discharge would have demonstrated improvements had they been analyzed at day 20.

The main limitation of our study is the relatively small number of patients included in this first interim analysis; therefore, further studies in larger populations are warranted, as also suggested by the high confidence interval (CI) around L-arginine in Tables 2 and 3. Additionally, we did not demonstrate that L-arginine administration actually increased arginine availability and we do not have data on respiratory rates and weight evolution. Of note, the enrollment took place in a setting dedicated to patients with severe forms of COVID-19; since all patients were hospitalized because of severe COVID-19, the generalizability of the results to patients with milder COVID-19 remains unclear. Some may argue that it might be easier for the sicker patients to transition to a lower level of respiratory support, potentially biasing the study towards the treatment arm; however, as shown in the stacked bar chart depicted in Fig. 2A, the main effects of L-arginine were especially evident in transitioning from HFNC to LTOT and from CPAP to HFNC.

Strengths of this trial included its blinded, placebo-controlled design, high adherence to the study protocol, rigorous monitoring for safety and adverse events, and rapid recruitment. Moreover, all patients were analyzed according to their randomization group, and follow-up was completed. We included patients with respiratory symptoms for up to 15 days prior to randomization while some trials of antiviral medications limit enrollment to patients with symptoms for a shorter duration, most likely in an effort to enrich the population for patients most likely to benefit.

Finally, it should be noted that both endothelial dysfunction and *T* cell impairment are consequences of low L-arginine bioavailability and contribute to complications of COVID-19, making a role for L-arginine supplementation biologically plausible. Thus, our results may have important clinical implications for COVID-19 treatment especially in low-resource environments were vaccination is not widely available given the safety and the affordability of oral L-arginine.

Taken together, the results of this interim analysis indicate that adding L-arginine to standard therapy in patients with severe COVID-19 significantly decreases the length of hospitalization and reduces the respiratory support assessed 10, but not 20, days after starting the treatment. The other secondary outcomes were not significantly different between groups.

## Declaration of Competing Interest

We declare no competing interests.

## Appendix Supplementary materials

Image, application 1

Image, application 2
