# Corrigendum to “Effects of adding L-arginine orally to standard therapy in patients with COVID-19: A randomized, double-blind, placebo-controlled, parallel-group trial. Results of the first interim analysis.”

**DOI:** 10.1016/j.eclinm.2022.101636

**Published:** 2022-09-13

**Authors:** Giuseppe Fiorentino, Antonietta Coppola, Raffaele Izzo, Anna Annunziata, Mariano Bernardo, Angela Lombardi, Valentina Trimarco, Gaetano Santulli, Bruno Trimarco

**Affiliations:** a COVID-19 Division, A.O.R.N. Ospedali dei Colli, Naples, Italy; b Department of Advanced Biomedical Sciences, “Federico II” University, Naples, Italy; c Department of Medicine, Fleischer Institute for Diabetes and Metabolsim (FIDAM), Einstein - Mount Sinai Diabetes Research Center (ES-DRC), Albert Einstein College of Medicine, New York, NY, USA; d Department of Microbiology and Immunology, Albert Einstein College of Medicine, New York, NY, USA; e Department of Neuroscience, Reproductive Sciences, and Dentistry, “Federico II” University, Naples, Italy; f Department of Molecular Pharmacology, Wilf Family Cardiovascular Research Institute, Einstein Institute for Aging Research, Institute for Neuroimmunology and Neuroinflammation, Albert Einstein College of Medicine, New York, NY, USA; g International Translational Research and Medical Education (ITME) Consortium, Naples, Italy

The authors wish to add the following information to their Research Paper:

In the original published version of this article, one of the affiliations for Prof. G. Santulli was incomplete; the correct affiliation is reported below: Department of Molecular Pharmacology, Wilf Family Cardiovascular Research Institute, Einstein Institute for Aging Research, Institute for Neuroimmunology and Inflammation, Albert Einstein College of Medicine, New York, NY, USA. Moreover, a declaration concerning the competing interest of one of the authors was not included. This issue has now been rectified; the corrected Declaration of Competing Interest can be found below.

Prof. B. Trimarco reports personal fees for speaker bureau, outside the content of the current manuscript, from Malesci, Damor, MSD, and Fidia.

The authors would like to apologise for any inconvenience caused.

