# Supplementary material for: Effects of adding L-arginine orally to standard therapy in patients with COVID-19: A randomized, double-blind, placebo-controlled, parallel-group trial. Results of the first interim analysis
Source: eClinicalMedicine. 2021 Sep 9;40:101125. doi: 10.1016/j.eclinm.2021.101125 (PMC8428476; doi:10.1016/j.eclinm.2021.101125)
Supplement: Supplementary file 1 [file mmc1.docx]

SUPPLEMENTARY MATERIAL

|  | HR | 95% CI | | p |
| --- | --- | --- | --- | --- |
|  |  | Lower | Upper |  |
| Age | 1.017 | 0.966 | 1.071 | 0.519 |
| Gender | 2.873 | 0.737 | 11.199 | 0.128 |
| Symptom duration | 0.616 | 0.196 | 1.940 | 0.408 |
| Hypertension | 0.905 | 0.265 | 3.095 | 0.874 |
| Diabetes | 0.028 | 0.001 | 0.608 | 0.023 |
| Creatinine | 0.866 | 0.306 | 2.449 | 0.786 |
| Lymphocytes | 1.088 | 0.988 | 1.198 | 0.085 |
| D-dimer | 1.001 | 1.000 | 1.001 | 0.072 |
| LMWH | 0.829 | 0.103 | 6.669 | 0.860 |
| Remdesivir | 1.121 | 0.339 | 3.709 | 0.852 |
| **L-arginine** | 4.443 | 1.290 | 15.301 | 0.018 |

**Table S1.**

Multivariable logistic regression analysis assessing the association of L-arginine with the primary outcome in the ITT population at 10 days. CI: confidence interval; LMWH: low molecular weight heparin; HR: hazard ratio; symptom duration before hospital admission was categorized as ≤8 and >8 days.

|  | HR | 95% CI | | p |
| --- | --- | --- | --- | --- |
|  |  | Lower | Upper |  |
| Age | 1.031 | 0.982 | 1.082 | 0.224 |
| Gender | 2.528 | 0.701 | 9.112 | 0.156 |
| Symptom duration | 0.536 | 0.172 | 1.665 | 0.281 |
| Hypertension | 1.960 | 0.580 | 6.630 | 0.279 |
| Diabetes | 0.511 | 0.079 | 3.283 | 0.479 |
| Creatinine | 0.641 | 0.237 | 1.735 | 0.381 |
| Lymphocytes | 1.014 | 0.934 | 1.101 | 0.739 |
| D-dimer | 1.000 | 0.999 | 1.001 | 0.440 |
| LMWH | 2.729 | 0.352 | 21.158 | 0.337 |
| Remdesivir | 2.640 | 0.769 | 9.064 | 0.123 |
| L-arginine | 2.104 | 0.671 | 6.595 | 0.202 |

**Table S2.**

Multivariable logistic regression analysis assessing the association of L-arginine with the primary outcome in the ITT population at 20 days. CI: confidence interval; LMWH: low molecular weight heparin; HR: hazard ratio; symptom duration before hospital admission was categorized as ≤8 and >8 days.
