# Supplementary material for: Effects of adding L-arginine orally to standard therapy in patients with COVID-19: A randomized, double-blind, placebo-controlled, parallel-group trial. Results of the first interim analysis
Source: eClinicalMedicine. 2021 Sep 9;40:101125. doi: 10.1016/j.eclinm.2021.101125 (PMC8428476; doi:10.1016/j.eclinm.2021.101125)
Supplement: Supplementary file 2 [file mmc2.pdf]

Short and long-term effects of adding L-arginine orally to standard therapy in patients with COVID-19. Randomized, parallel group, double-blind placebo-controlled clinical study

Dr. Giuseppe Fiorentino, Prof. Bruno Trimarco
COVID-19 Division Colli Hospital (Cotugno)

Index

**1. Rationale**	**page 3**
**2. Aim**	**page 4**
**3. Endpoints**	**page 4**
**4. Study design**	**page 4**
**5. Enrollment**	**page 5**
**6. Inclusion criteria**	**page 5**
**7. Exclusion criteria**	**page 5**
**8. Data Collection Sheet**	**page 6**
**9. Evaluation tools**	**page 6**
**Sample size**	**page 7**
**Statistical Analysis**	**page 7**
**13. Study phases**	**page 8**
**14. Data protection and patient confidentiality**	**page 8**
**References**	**page 9**

1. Rationale

The COVID-19 pandemic with over 30,000,000 million infected people and 1,000,000 deaths worldwide represents a health emergency for which a vaccine is not yet available. It is therefore crucial to identify the pathogenetic mechanisms of this disease in order to set up an effective therapeutic strategy. The studies conducted so far have shown that the infection determines the involvement of many organs and that endothelial dysfunction plays a central role in its pathogenesis (1). This observation also explains why elderly subjects, patients with diabetes mellitus and those suffering from arterial hypertension, who have an altered endothelial function as their common denominator, have a worse prognosis when they contract COVID-19 infection. On the other hand, it is well known that the endothelium is able to modulate platelet aggregation and coagulation processes, contributes to innate immunity by reducing inflammation, as well as regulating vascular resistance and protecting tissues from the harmful effect of free radicals of oxygen through the production of antioxidants. It is therefore not surprising that endothelial dysfunction induced by viral infection involves thromboembolic phenomena at the level of vital organs such as the brain, heart and, above all, the lung or inflammatory diseases such as pneumonia or myocarditis (2).

It is also important to note that according to the COVID Symptom Study, which enrolled more than 4 million people cured of COVID-19 infection in the United States of America, the United Kingdom and Sweden, a COVID-19 syndrome has been defined. post-acute, characterized by the persistence of symptoms for up to three weeks from onset, and a chronic one that extends beyond 12 weeks (3). These syndromes not only affect patients in whom the infection has had a more severe course (4) and are characterized, in addition to asthenia and dyspnea, by typical symptoms of pathology of specific organs such as the lungs, brain, kidney and the heart (5). More than the persistence of the virus, these complications appear to be related to endothelial dysfunction with a cytokine storm that sustains severe inflammation, and the state of hypercoagulability (6,7).

Therefore, it is crucial to improve or preserve endothelial function (8). This goal can be achieved through the supplementation of arginine, the key amino acid for the production of nitric oxide and citrulline by the endothelial cell (8). In fact, the administration of this supplement increases the urinary excretion of nitrates in subjects with endothelial dysfunction (9) and induces an improvement in physical performance both in athletes (10) and in patients with heart failure (11). Preliminary experiences conducted in patients affected by COVID-19 infection by adding the daily oral administration of two bottles of Bioarginine FarmaDamor to standard therapy have shown favorable effects on discharge times, on recovery of lymphocyte number and on the P\F ratio between pO_2 and FiO_2. In particular, for the latter parameter there is almost a doubling of the recovery speed.

2. Aim

Based on preliminary experiences, it was found that the addition of two bottles of Bioarginine FarmaDamor to the standard therapy showed various favorable effects. The purpose of this study is to understand whether the addition of two bottles of Bioarginine FarmaDamor orally to standard therapy can have favorable effects on discharge times, recovery of lymphocytes and the P\F ratio.

3. Endpoints

Primary Endpoint:

- Reduction of P / F normalization times

Secondary Endpoints:

- Reduction of lymphocyte normalization times
- Reduction of hospitalization times
- Reduction of the negative time of the RT-PCR for SARS-CoV2 on nasopharyngeal swab
- Incidence of post-acute COVID-19
- Incidence of chronic COVID-19

4. Study design

Spontaneous, single-center study with a parallel group scheme, double-blind randomized, placebo-controlled to evaluate whether the addition of two bottles of BioArginine FarmaDamor orally to our hospital standard therapy in subjects affected by COVID-19 is useful for the treatment of this disease.

5. Enrollment

We will need to enroll 290 COVID-19 patients who will be given 2 bottles of Bioarginine Damor orally in addition to the standard therapy that will be compared with a historical group of patients a historical group of COVID-19 patients undergoing the same therapeutic treatment except the supplementation of 2 oral bottles of BioArginina Farmadamor per day. Number of patients needed: 290 COVID-19 patients.

6. Inclusion criteria:

• age> 18 years
• Diagnosis of COVID-19, confirmed by RT-PCR on a nasopharyngeal swab
• COVID-19 pneumonia with the following clinical characteristics: SpO2 in ambient air <93% and Alveolar oxygen pressure / inspiratory oxygen fraction (PaO2 / FiO2 - P/F <300 mmHg.
• Lymphocytopenia defined as lymphocytes <1500 / mcL or <20% of white blood cells

7. Exclusion criteria

• History of L-arginine intolerance
• Severe chronic pulmonary disease
• Pregnancy or breastfeeding
• Neutropenia due to neoplasms of the haematopoietic system or other organs with invasion of the bone marrow
• Use of non-steroidal immunosuppressive drugs or cytotoxic chemotherapies within the previous three weeks
• Refusal to give consent to participate in the study

8. Procedures

Eligible patients will be assigned, with a 1: 1 ratio, based on a randomization table elaborated on the computer, using the random function of excel, to add to the standard therapy of our hospital twice a day orally 1 bottle of Bio-Arginina FarmaDamor or 1 bottle of identical appearance containing equal quantities of physiological solution, for the entire duration of hospitalization. Both products will be made available free of charge by Farmadamor and will be in completely identical packaging so as not to allow them to be distinguished. The boxes containing the individual bottles will be marked with the initials "treatment A" or "treatment B" and the key will be contained in a sealed envelope delivered to the Principal Investigator which must not be opened before the completion of the study except for any emergencies.
In this way, the patient, the ward doctors and those who will have to evaluate the outcome of the therapy will not be aware of the type of supplementation provided. All clinical interventions, such as the use of steroids, antibiotics, ventilation strategy, laboratory investigations, etc. will be at the discretion of the treating physicians for both treatment groups according to clinical needs.
During the entire duration of the study, the appearance of any adverse events and their severity and the procedures implemented until the suspension of treatment with bioarginine will be detected.

9. Data Collection Form

The study includes an Electronic Data Collection Card. The study investigators will fill out a data sheet for each patient enrolled in the study.
Upon admission, the following parameters were assessed:
• Time between the onset of symptoms and hospitalization;
• Symptoms and signs (fever, cough, sputum, dyspnea, asthenia);
• Smoking habit (smoker, ex-smoker, non-smoker);
• Concomitant diseases (arterial hypertension, diabetes mellitus, heart disease, obesity);
• Use of antihypertensive drugs / ACE inhibitors or ATII antagonists);
• SARS-CoV-2 RNA RT-PCR detection on nasopharyngeal swab;
• IgM and IgG anti-SARS-CoV2;

Evaluation tools

The physician must record on the data collection card all the assessments present in the medical record according to the scheme provided for by the data collection card.
It should be emphasized that all the parameters in the data collection card are included in the records present in the medical records of COVID-19 patients.

10. Sample size

There are no reliable data available for an accurate calculation of the sample size, so we used the data of preliminary clinical experiences conducted with the administration of L-Arginine in patients with COVID-19 comparing them with those of a historical group of COVID-19 patients undergoing at the same therapeutic treatment except the supplementation of 2 oral bottles of BioArginina Farmadamor per day. On the basis of these data we hypothesized a 2-side alpha level of 0.05 and a power of 80% to detect a difference of 3 days in advance of the normalization of the P/F between the two groups under study, assuming that this value constitutes a reduction of the 35% compared to the control group. Based on these hypotheses it is necessary to enroll a population of 290 patients.

11. Statistical Analysis

Two interim analyzes are planned for the evaluation of efficacy and tolerability after completion of the first 100 and 200 patients. The study suspension threshold is set at $p<0.001$ for efficacy and $p<0.01$ for tolerability.
Student's t test for independent samples will be used to evaluate any differences in all continuous variables between the BioArginine and placebo group at each phase of the study, while the $\chi 2$ test will be used to compare categorical variables. The two-way analysis of variance for repeated measures on two factors (2x2; condition vs time) will be used to evaluate differences in plasma concentrations of the parameters taken into consideration between treatment with Arginine and that with Placebo. All analyses will be performed using SPSS 25. The level of statistical significance will be set at a value of $p\leq 0.05$.

Study phases

The study will include the recording of clinical, laboratory and instrumental data for each patient on a daily basis, from the date of admission to the date of discharge. It is estimated that the data will be recorded for an expected period of no more than 3 months, even assuming longer periods.

12. Data protection and patient confidentiality

Investigators and staff involved in this trial will comply with the Fundamental Principles of Good Clinical Practice (GCP) regarding the collection, storage, processing and disclosure of personal information. All data will be stored under the responsibility of the principal investigator and study coordinator and will be shared only anonymously.

References

1 Libby, P, Lüscher. COVID-19 is, in the end, an endothelial disease. *European Heart Journal*, 2020, *41*(32).

2 Sardu C, Gambardella J, Morelli MB et al. Hypetension, Thrombosis, Kidney Failure, and Diabetes: Is COVID-19 an Endothelial Disease? A Comprehensive Evaluation of Clinical and Basic Evidence. J Clin Med. 2020; 9(5):1417

3) Greenhalgh T, Knight M, A'Court C, Buxton M, Husain L. Management of post-acute Covid-19 in primary care. *BMJ*. 2020;370:m3026.

4) Tenforde MW, Kim SS, Lindsell CJ, et al; IVY Network Investigators; CDC COVID-19 Response Team; IVY Network Investigators. Symptom duration and risk factors for delayed return to usual health among outpatients with COVID-19 in a multistate health care systems network: United States, March-June 2020. *MMWR Morb Mortal Wkly Rep*. 2020;69(30):993-998.

5) Carfì A, Bernabei R, Landi F; Gemelli Against COVID-19 Post-Acute Care Study Group. Persistent symptoms in patients after acute COVID-19. *JAMA*. 2020;324(6):603-605.

6) Puntmann VO, Carerj ML, Wieters I, et al. Outcomes of cardiovascular magnetic resonance imaging in patients recently recovered from coronavirus disease 2019 (COVID-19). *JAMA Cardiol*.

7) Rajpal S, Tong MS, Borchers J, et al. Cardiovascular magnetic resonance findings in competitive athletes recovering from COVID-19 infection. *JAMA Cardiol*. Published online September 11, 2020.

8) Gambardella J, Khondar W, Morelli MB, et al. Arginine and Endothelial function. *Biomedicines* 2020, *8*(8), 277.

9) Boger RH, Bode-Boger SM, Szuba A, et al. Asymmetric Dimethylarginine (ADMA): A Novel Risk Factor for Endothelial Dysfunction. Circulation 1998;98:1842

10) Suzuki I, Sakuraba K, Horiike T, et al. A combination of oral L-citrulline and L-arinine improved 10-min full power cycling test in male collegiate soccer players: a randomized cross-over trial. Eur J Appl Physiol 2019; 119: 1075

11) Rector TS, Bank AJ, Mullen KA, et al. Randomized, double-blind, Placebo-Controlled Study of Supplemetal Oral L-Arginine in Patients With Heart Failure. Circulation. 1996; 93: 2135

12) del Rio C, Collins LF, Malani P. Long-term Health Consequences of COVID-19. JAMA Published online October 5,2020.
